# Comparison of semi-quantitative and quantitative dynamic contrast-enhanced MRI evaluations of vertebral marrow perfusion in a rat osteoporosis model

**DOI:** 10.1186/s12891-017-1800-1

**Published:** 2017-11-14

**Authors:** Jingqi Zhu, Zuogang Xiong, Jiulong Zhang, Yuyou Qiu, Ting Hua, Guangyu Tang

**Affiliations:** 1 Department of Radiology, Shanghai Tenth People’s Hospital, Tongji University School of Medicine, 301 Middle Yanchang Road, Shanghai, 200072 China; 2 Department of Radiology, East Hospital, Tongji University School of Medicine, Shanghai, 200120 China

**Keywords:** Osteoporosis, Dynamic contrast-enhanced magnetic resonance imaging, Micro-computed tomography, Vascular endothelial cell, Microvessel density

## Abstract

**Background:**

This study aims to investigate the technical feasibility of semi-quantitative and quantitative dynamic contrast-enhanced magnetic resonance imaging (DCE-MRI) in the assessment of longitudinal changes of marrow perfusion in a rat osteoporosis model, using bone mineral density (BMD) measured by micro-computed tomography (micro-CT) and histopathology as the gold standards.

**Methods:**

Fifty rats were randomly assigned to the control group (*n*=25) and ovariectomy (OVX) group whose bilateral ovaries were excised (*n*=25). Semi-quantitative and quantitative DCE-MRI, micro-CT, and histopathological examinations were performed on lumbar vertebrae at baseline and 3, 6, 9, and 12 weeks after operation. The differences between the two groups in terms of semi-quantitative DCE-MRI parameter (maximum enhancement, E_max_), quantitative DCE-MRI parameters (volume transfer constant, K^trans^; interstitial volume, V_e_; and efflux rate constant, K_ep_), micro-CT parameter (BMD), and histopathological parameter (microvessel density, MVD) were compared at each of the time points using an independent-sample *t* test. The differences in these parameters between baseline and other time points in each group were assessed via Bonferroni’s multiple comparison test. A Pearson correlation analysis was applied to assess the relationships between DCE-MRI, micro-CT, and histopathological parameters.

**Results:**

In the OVX group, the E_max_ values decreased significantly compared with those of the control group at weeks 6 and 9 (*p*=0.003 and 0.004, respectively). The K^trans^ values decreased significantly compared with those of the control group from week 3 (*p*<0.05). However, the V_e_ values decreased significantly only at week 9 (*p*=0.032), and no difference in the K_ep_ was found between two groups. The BMD values of the OVX group decreased significantly compared with those of the control group from week 3 (*p*<0.05). Transmission electron microscopy showed tighter gaps between vascular endothelial cells with swollen mitochondria in the OVX group from week 3. The MVD values of the OVX group decreased significantly compared with those of the control group only at week 12 (*p*=0.023). A weak positive correlation of E_max_ and a strong positive correlation of K^trans^ with MVD were found.

**Conclusions:**

Compared with semi-quantitative DCE-MRI, the quantitative DCE-MRI parameter K^trans^ is a more sensitive and accurate index for detecting early reduced perfusion in osteoporotic bone.

## Background

Osteoporosis (OP) is a chronic disorder, leading to an increased risk of fragile fractures. The pathophysiology of OP includes hormonal, microenvironmental, and genetic determinants which have been associated with a misbalance between bone formation and resorption.

Recently, a hypothetic pathophysiological mechanism for OP has been proposed involving reduced perfusion within the bone marrow which may affect the bone marrow microenvironment [1, 2]. More evidence from dynamic contrast-enhanced magnetic resonance imaging (DCE-MRI) indicates that compromised perfusion could be deleterious to the bone marrow [1–4]. However, the value of different DCE-MRI approaches such as semi-quantitative and quantitative analyses to assess the microcirculation in OP is unknown.

In this study, we aimed to compare the performances between semi-quantitative and quantitative DCE-MRI in the evaluation of bone marrow blood perfusion in a rat OP model after ovariectomy (OVX), regarding micro-computed tomography (micro-CT) and histopathological results as a referential gold standard. The effects of two techniques on early diagnosis of OP were also evaluated.

## Methods

### Animals

Fifty three-month-old female Sprague Dawley rats (weight, 250 to 290 g; Department of Laboratory Animal Science, Tongji University, Shanghai, China) were used in our study. Each cage housed five rats at 20 °C to 25 °C, with a 12 h light–dark cycle. Standard laboratory rat diet and water were available ad libitum. The experiment was approved by the animal review committee of Shanghai Tenth People’s Hospital of Tongji University and the ethics committee of Science and Technology Commission of Shanghai Municipality [SYXK (Shanghai) 2011–0111], and was strictly in accordance with the guidelines for the care and use of laboratory animals as established by the Department of Science and Technology of China in 2006.

### Rat OP model

Surgery was performed when all rats had been acclimatized to the new conditions for a week. The rats were randomly divided into a control group (*n* = 25) and OVX group (*n* = 25). All animals underwent operations while anesthetized by an intraperitoneal injection of 4% chloral hydrate (10 ml/kg). The rats in the control group underwent sham operation, defined as exteriorization but not removal of the ovaries. The OVX group underwent a bilateral OVX operation. After surgery, the two groups were fed a standard laboratory diets. Only two rats died in the process of conducting this study. One rat in the control group died of abdominal hemorrhage after the operation, whereas the other rat in the OVX group died of an anesthesia overdose at week 12. Hence, an additional two rats were provided to the corresponding groups as replacements. Each rat was weighted before surgery and the MRI scan. DCE-MRI and micro-CT examinations were performed at 0 (baseline), 3, 6, 9, and 12 weeks post operation on two groups of rats (five rats at each time point in each group).

### DCE-MRI examination

DCE-MRI examination was performed on a 3.0-T MRI scanner (Magnetom Verio; Siemens Medical Solutions, Erlangen, Germany) with a gradient strength of 40 mT/m and a gradient slew rate of 200 mT/ms. A body coil was employed to transmit radio frequency signals. A small animal coil (C-MUC18-H300-AS; Shanghai Chenguang Medical Technology Co., Ltd., Shanghai, China) was used to receive signals.

Following a coronal scout scan, sagittal T1-weighted images of the lumbar vertebrae were obtained using the following protocol: three-dimensional volumetric interpolated breath-hold examination sequence; repetition time (msec)/echo time (msec), 7.1/2.05; field of view, 180 mm; slice thickness, 1.5 mm; averages, 1; flip angles, 5° and 15°; matrix, 69 × 192; and pixel size, 1.3 mm × 0.9 mm. Once the baseline scan was finished, a bolus of gadopentetate dimeglumine (Magnevist, Bayer Schering, Berlin, Germany; concentration, 0.5 mol/L) diluted in 0.9% saline to a final concentration of 0.06 mol/L was rapidly injected manually (injection time, 1–2 s; dose, 0.3 mmol/kg of body weight) into the tail vein through a 24-gauge intravenous catheter. Overall, the DCE-MRI scan required a total acquisition time of 493 s to acquire 592 dynamic images.

The DCE-MRI analysis was processed on an imaging workstation (Tissue 4D, Syngo multimodality workplace, software version B17_43.1_1.0, Siemens Healthcare). A region of interest (ROI) was drawn along the vertebral body of the fifth lumbar vertebra (L5), excluding the vertebral cortex. The signal intensity values in the locations of the ROI were plotted against time as time–signal intensity curve (TIC). One perfusion index of the semi-quantitative DCE-MRI analysis, maximum enhancement (E_max_), defined as the maximum percentage increase in signal intensity from baseline, was acquired from the TIC according to the following eq. [1]: ${E}_{\mathrm{max}}=\frac{I_{\mathrm{max}}-{I}_{base}}{I_{base}}\times 100\%$, where *I*_max_ was defined as the peak signal intensity of the TIC, and *I*_*base*_ was calculated as the mean signal intensity of the baseline images. Three quantitative DCE-MRI indexes, K^trans^, V_e_, and K_ep_, were calculated by arterial input function based on the Tofts model according to the following eqs. [5]:

$$ {K}^{trans}={V}_e\times {K}_{ep}, $$

$$ {C}_t={V}_e\times {C}_e, $$

_and_ $$ \frac{dC_t}{dt}={K}^{trans}{C}_p-{K}^{trans}{C}_t/{V}_e $$_,_

where *C*_*t*_, *C*_*e*_, and *C*_*p*_ are the concentrations of the contrast agent in the tissue, extravascular-extracellular space, and plasma, respectively. All DCE-MRI indexes were measured twice and the average values were taken.

### Micro-CT examination

The rats were sacrificed with an overdose of 4% chloral hydrate (20 ml/kg) via an intraperitoneal injection after MRI examination. The L5 vertebrae were dissected, stored in 4% paraformaldehyde, and preserved at 4 °C for micro-CT (Explore locus, GE healthcare, Milwaukee, USA) examination. Each vertebra was imaged with the following protocol: tube voltage, 80 kV; tube current, 450 μA; exposure time, 400 ms; rotation step, 0.5°, a rotation of 360°; detector bin mode, 2 × 2; voxel size, 45 μm × 45 μm × 45 μm; frame averaging, 1; and scan time, 25 min. Cone beam reconstruction was performed on the projected files to acquire 4000 pixel × 4000 pixel two-dimensional images. Ring artifact correction, smoothing, and beam hardening correction were set at 10%, 1%, and 7%, respectively. Gaussian filtration (σ = 0.8, support = 1) was used to reduce signal noise and to maintain a sharp contrast between bone and marrow. A threshold of 800 was defined to isolate bone tissue. Axial, coronal, and sagittal micro-CT images showed that the ROI was a cuboid containing the cancellous bone 0.3 mm distal to the endplate within the L5 vertebral body, which was similar in size to that in the DCE-MRI to ensure that both ROIs matched. The bone mineral density (BMD, milligrams per cubic centimeter of trabeculae) value was calculated with GE Healthcare Microview V.2.1.1 software. Each L5 vertebral body was examined twice. The average values were taken as the final data.

### Transmission electron microscopy (TEM) observation

One rat in the control group and two rats in the OVX group were randomly selected for TEM observation at each time point. The excised L4 vertebral body was cut into several pieces, quickly fixed in a mixture of 3% glutaraldehyde and 4% paraformaldehyde at 4 °C for one day, and decalcified in 4% ethylenediaminetetraacetic acid for 2–3 weeks at room temperature. Decalcified tissue was washed with 0.1 mol/L phosphate buffered saline (pH = 7.0), fixed in 1% osmium tetroxide, dehydrated through a series of ascending ethanol solutions, embedded in epoxy resin E51, and sectioned for double staining with 3% uranyl acetate and lead citrate. The mitochondria of vascular endothelial cells (VEC) and the gap between VECs in the bone marrow sample were observed using TEM (JEM-1230, JEOL, Tokyo, Japan).

### Microvessel density (MVD) assessment

The L5 vertebral body was fixed in 10% buffered formalin for one day after micro-CT examination and decalcified in 4% ethylenediaminetetraacetic acid for 2–3 weeks. The decalcified sample was dehydrated in ethanol, embedded in paraffin, and cut into 4 μm-thick sections. Sections were dewaxed, microwaved, and rehydrated. Endogenous peroxidase and non-specific binding activity were blocked by incubation with 3% hydrogen peroxide and non-immune goat serum, respectively. VECs were stained with a rabbit-anti-rat CD34 (Wuhan Boster Biological Engineering Co., Ltd., Hubei, China; dilution 1:100). The immunochemical analysis was performed by an experienced pathologist without knowledge of the group allocation status. Three sections (the top, middle, and bottom levels of the specimen; eight 200× fields in hot spots per section) per vertebral body were selected for MVD assessment using a Leica Q-win Plus image analysis system (Leica Microsystems, Wetzlar, Germany). Care was taken to identify angiogenic CD34 positive VECs and to not count CD34-positive hematopoietic cells. The MVD value was determined as the mean microvessel number measured from three sections in each vertebra.

### Statistical analysis

All statistical analyses were performed with SPSS 19.0 software (SPSS Inc., Chicago, IL, USA). Data were expressed as the means ± standard deviations. The differences between the two groups in terms of semi-quantitative DCE-MRI parameter (E_max_), quantitative DCE-MRI parameters (K^trans^, V_e_, and K_ep_), micro-CT parameter (BMD) and histopathological parameter (MVD) at the same time point were compared by using an independent-sample *t* test. The differences in those variables between baseline and the other time points in each group were assessed via Bonferroni’s multiple comparison test. A Pearson correlation analysis was applied to assess the relationships between BMD, MVD, and DCE-MRI parameters. A *p* value <0.05 was considered to be statistical significance.

## Results

### Semi-quantitative DCE-MRI analysis

At baseline, no significant difference was found in the E_max_ between two groups (*p* = 1.000). The E_max_ values did not differ statistically over five time points in the two groups (*p* > 0.05 for all). Although the E_max_ values of the OVX group decreased compared with that of the control group from week 3 on, the only significant differences were at weeks 6 and 9 (*p* values were 0.003 and 0.004, respectively, for weeks 6 and 9).

### Quantitative DCE-MRI analysis

At baseline, there was no significant difference in K^trans^, V_e_, and K_ep_ between the two groups (*p* > 0.05 for all). In the control group, these parameters did not differ statistically across the five time points (*p* > 0.05 for all). In contrast, in the OVX group, the K^trans^ values decreased significantly compared with those at baseline (*p* < 0.001 for all) and those of the control group at the same time points from week 3 on (*p* values were 0.029, 0.027, 0.039, and 0.041, respectively, for weeks 3, 6, 9, and 12). From week 3 on, the V_e_ values of the OVX group decreased compared with those at baseline and those of the control group. However, the difference reached statistical significance only at week 9 (*p* = 0.018 versus baseline, *p* = 0.032 versus control group at the same time point). The K_ep_ values of the OVX group did not differ statistically compared with those of the control group at the same time point (*p* > 0.05 for all). No significant differences were found for K_ep_ among the different time points in the OVX group (*p* > 0.05 for all). A comparison of semi-quantitative and quantitative DCE-MRI analysis between the two groups is shown in detail in Table 1.

**Table 1:** DCE-MRI parameters of L5 vertebral bodies at five time points

Time point	Control group	OVX group	*t* value	*P* value
E_max_				
Baseline	1.51 ± 0.19	1.51 ± 0.06	0.000	1.000
Week 3	1.53 ± 0.34	1.35 ± 0.14	1.053	0.323
Week 6	1.69 ± 0.19	1.30 ± 0.09	4.182	0.003^#^
Week 9	1.58 ± 0.09	1.26 ± 0.16	3.971	0.004^#^
Week 12	1.39 ± 0.16	1.35 ± 0.16	0.313	0.762
K^trans^ (min^−1^)				
Baseline	0.29 ± 0.06	0.34 ± 0.03	−1.653	0.137
Week 3	0.28 ± 0.08	0.18 ± 0.03^*^	2.646	0.029^#^
Week 6	0.29 ± 0.07	0.20 ± 0.02^*^	2.691	0.027^#^
Week 9	0.26 ± 0.08	0.18 ± 0.01^*^	2.462	0.039^#^
Week 12	0.25 ± 0.06	0.16 ± 0.02^*^	2.724	0.041^#^
V_e_ (mL/100 mL)				
Baseline	0.36 ± 0.08	0.38 ± 0.09	−0.376	0.716
Week 3	0.41 ± 0.18	0.25 ± 0.07	1.825	0.105
Week 6	0.37 ± 0.08	0.29 ± 0.05	1.825	0.105
Week 9	0.33 ± 0.08	0.22 ± 0.05^*^	2.600	0.032^#^
Week 12	0.27 ± 0.05	0.25 ± 0.06	0.556	0.594
K_ep_ (min^−1^)				
Baseline	0.79 ± 0.08	0.94 ± 0.18	−1.667	0.134
Week 3	0.70 ± 0.29	0.84 ± 0.22	−0.868	0.410
Week 6	0.77 ± 0.15	0.71 ± 0.18	0.560	0.591
Week 9	0.80 ± 0.23	0.82 ± 0.24	−0.194	0.851
Week 12	0.86 ± 0.16	0.65 ± 0.15	2.224	0.057

Data are expressed as the means ± standard deviations. OVX group represents the rats underwent bilateral ovariectomy. Control group represents the rats underwent sham operation

^*^ *P* < 0 .05, versus baseline in the same group

^#^ *P* < 0.05, control group versus OVX group at the same time point

### Micro-CT analysis

**Fig. 1:**
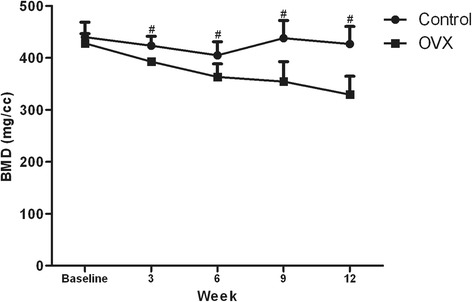
Graph of BMD data in L5 vertebral bodies for the control and OVX groups. Data are expressed as the means ± standard deviations. ^#^ *P* < 0.05, control group versus OVX group at the same time point

**Fig. 2:**
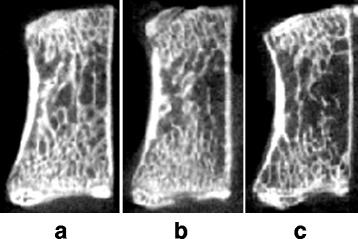
Midsagittal micro-CT images of L5 vertebral bodies in the OVX group. The images show normal cancellous bone architecture at baseline (**a**) and continuously microarchitectural deterioration from week 3 (**b**) to week 12 (**c**)

At baseline, there was no significant difference in BMD between the two groups (*p* = 0.453). In the control group, no significant difference in BMD was seen among the different time points (*p* > 0.05 for all). The BMD values of the OVX group decreased significantly compared with those of the baseline from week 6 on (*p* values were 0.013, 0.004, and <0.001, respectively, for weeks 6, 9, and 12) and compared with those of the control group at the same time points from week 3 on (*p* values were 0.008, 0.032, 0.007, and 0.002, respectively, for weeks 3, 6, 9, and 12) (Figs. 1 and 2).

### TEM observation

**Fig. 3:**
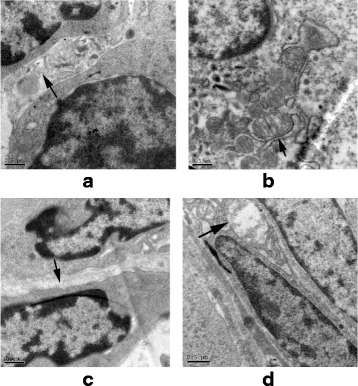
TEM sections of L4 vertebral bodies show (**a**) loose gap between VECs (*arrow*) with (**b**) normal mitochondria (*arrow*) in the control group, and enhanced vasoconstriction appeared as (**c**) tight gap between VECs (*arrow*) with (**d**) swollen mitochondria (*arrow*) at week 3 after OVX (original magnification ×20,000)

In the control group, loose gaps between VECs with normal mitochondria were observed over five time points (Fig. 3a and b). However, tighter gaps between VECs with swollen mitochondria were seen in the OVX group compared to those in the control group from week 3 on (Fig. 3c and d).

### MVD assessment

**Fig. 4:**
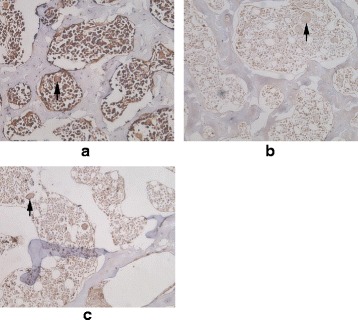
Decalcified histopathologic sections of L5 vertebral bodies in the OVX group using endothelial marker CD34 stain (original magnification ×200). Microvessels (*arrows*) remain constant at baseline (**a**) and week 3 (**b**), and decrease at week 12 (**c**) after OVX operation

There was no significant difference in MVD between the two groups at baseline (*p* = 0.230). In the control group, the MVD values remained stable throughout the observation period (*p* > 0.05 for all). At week 12, the MVD values of the OVX group decreased significantly compared with those of the baseline (*p* = 0.005) and those of the control group at the same time point (*p* = 0.023) (Table 2, Fig. 4).

**Table 2:** MVD of L5 vertebral bodies at five time points

Time point	Control group	OVX group	*t* value	*P* value
Baseline	4.80 ± 0.52	4.25 ± 0.80	1.299	0.230
Week 3	4.05 ± 0.49	3.84 ± 0.48	0.699	0.504
Week 6	4.29 ± 0.54	4.24 ± 0.33	0.183	0.859
Week 9	4.27 ± 0.60	3.52 ± 0.84	1.629	0.142
Week 12	3.79 ± 0.77	2.58 ± 0.57^*^	2.817	0.023^#^

Data are expressed as the means ± standard deviations. OVX group represents the rats underwent bilateral ovariectomy. Control group represents the rats underwent sham operation

^*^ *P* < 0 .05, versus baseline in the same group

^#^ *P* < 0.05, control group versus OVX group at the same time point

### Correlations between BMD, MVD, and DCE-MRI parameters

There were significant positive correlations of BMD with E_max_ and K^trans^ (*r* = 0.448, *p* = 0.001; *r* = 0.414, *p* = 0.003, respectively). However, there were no correlations of BMD with V_e_ and K_ep_ (*r* = 0.267, *p* = 0.061; *r* = 0.182, *p* = 0.205, respectively). A weak positive correlation between MVD and E_max_ was found (*r* = 0.289, *p* = 0.042). However, there were strong positive correlations of MVD with K^trans^ and V_e_ (*r* = 0.399, *p* = 0.004; *r* = 0.379, *p* = 0.007, respectively). The relationship between MVD and K_ep_ was not significant (*r* = −0.035, *p* = 0.811) (Table 3).

**Table 3:** Correlations between BMD, MVD, and DCE-MRI parameters

DCE-MRI parameters	BMD		MVD	
	*r*	*P*	*r*	*P*
E_max_	0.448	**0.001**	0.289	**0.042**
K^trans^	0.414	**0.003**	0.399	**0.004**
V_e_	0.267	0.061	0.379	**0.007**
K_ep_	0.182	0.205	−0.035	0.811

Bold fonts indicated statistical difference

*BMD* bone mineral density, *MVD* microvessel density

## Discussion

OP is a significant public health problem that is characterized by a systemic impairment of bone mass and microarchitecture. This condition is most commonly reported among postmenopausal women. Bone loss in the OVX rat, which shares striking similarities with postmenopausal bone loss in aged women, is considered the “gold standard” animal model in postmenopausal OP studies [6]. Radiological assessment is mostly used in the detection of OP to prevent fragile fractures. However, BMD alone cannot predict fracture risk reliably or earlier [7]. Therefore, other aspects which may reflect the bone microenvironment have been investigated in the last two decades.

Recently, a few studies have proposed the hypothesis that reduced bone perfusion is closely related to compromised BMD [1, 4, 8–10]. Our longitudinal observation showed a tendency of a decrease in the semi-quantitative perfusion parameter E_max_ accompanied with a decrease in BMD in the OVX group from week 6 on, which is consistent with other reports [1, 9]. Semi-quantitative DCE-MRI analysis consists of a group of parameters such as E_max_ and enhancement slope that require calculation based on TIC, which allows for noninvasive evaluation of marrow perfusion in OP [1, 4, 11, 12]. Nonetheless, semi-quantitative DCE-MRI is more influenced by individual hemodynamic fluctuations and MRI protocols, and its hemodynamic parameters lacks a clear interpretation related to the underlying physiology [13, 14].

In the past decade, quantitative DCE-MRI parameters have been used to successfully reflect histological changes in vasculature. It is because of developments in pharmacokinetic models have enabled them to resolve those problems for semi-quantitative DCE-MRI [12, 15]. Therefore, a direct relationship between quantitative DCE-MRI parameters and bone marrow perfusion can be established. K^trans^ represents the volume transfer constant from the plasma space to the interstitial space. This physiological parameter is fully determined by plasma flow and the permeability-surface area product. K_ep,_ known as the interstitium-to-plasma rate constant, reflects the reverse transport of gadolinium back into the vascular space. V_e_ measures the interstitial volume, which is defined as the extravascular-extracellular volume fraction [15].

To the best of our knowledge, few longitudinal animal-based studies have compared semi-quantitative with quantitative DCE-MRI to determine which better reflects the perfusion condition in OP. In our study, acute estrogen deficiency caused a significant decrease in K^trans^ of the bone marrow from week 3 after OVX. It was identified by TEM observation, which showed vascular endothelial dysfunction appearing as tighter gaps between VECs with swollen mitochondria. Gulhan et al. [16] reported that postmenopausal women with OP may have an association with higher endothelin-1levels than those without OP. Animal studies indicated that the tendency of increased endothelin-1 serum levels in the OVX rats was one of the most likely causes of enhanced vasoconstriction and decreased permeability [2]. These findings strongly support the hypothesis that vascular endothelial dysfunction after OVX induced low bone marrow perfusion at an early stage [1]. It is notable that no significant change on MVD calculation was found between the OVX and control group until week 12. This phenomenon implies that the decrease in K^trans^ of the OVX group in the late stage may be attributed to the decrease of MVD in the bone marrow. Our study also found that the V_e_ values of the OVX group decreased significantly only at week 9 compared with those of the control group. It is obvious that the change of V_e_ values was later than that of the K^trans^ values in the OVX group. The increased and enlarged fat cells in the bone marrow of OVX rat occupied the trabecular space, which induced a reduction in extravascular-extracellular space [1, 17, 18]. This indicates that microvascular dysfunction may occur earlier than the accumulation of fat cells. K_ep_, as the ratio of K^trans^ to V_e_, was not significantly different throughout the observation period, which may be due to the reduction of K^trans^ and V_e_ in synchrony. Ma et al. [19] found that the two pharmacokinetic parameters (K^trans^ and V_e_) showed a significant decrease in OP patients compared to normal subjects. In a few longitudinal animal-based studies, K^trans^ was reported to be a promising parameter to monitor acute ischemia in osteoporotic bone [2], while K_ep_ may be a sensitive index to reflect long-term chronic ischemia in OP due to vessel rarefication and maturation in the bone marrow [3]. Our results are consistent with these reports, which indicated that reduced bone marrow perfusion of OP could be directly reflected by quantitative DCE-MRI. However, no difference in E_max_ was found between the two groups until week 6 after the operation and the correlation coefficient of K^trans^ was the highest between MVD and DCE-MRI parameters. Together, these findings suggest that K^trans^ changes earlier and is more precise than E_max_, although both of them have strong positive correlations with BMD and could reflect the reduced bone marrow perfusion in OP. In our study, DCE-MRI can be well performed on the vertebral body of the rat, which illustrates that this technique will be a feasible and reliable modality to quantify bone marrow perfusion in humans. Dual-energy X-ray absorptiometry (DXA) is a well-standardized, inexpensive, and convenient technique for BMD measurement, which has a low radiation dose. However, it is a two-dimensional measurement and is sensitive to degenerative diseases, which may alter the BMD value [20]. Compared with DXA, DCE-MRI is a relatively high-cost technique. However, lack of radiation and acquisition of compromised bone perfusion parameters in earlier stage of OP makes it more attractive in the clinical practice and more sensitive in monitoring the therapeutic effect. A comprehensive evaluation including BMD and bone metabolism should be performed for an OP patient regardless of whether it is for a precise diagnosis or the assessment of efficacy.

Our study has several limitations. First, the number of rats at each time point was relatively small, but it conforms to the statistical regulation. Experimental errors may affect the results because of individual differences. Second, only the L5 vertebral body was studied by DCE-MRI, micro-CT examination and immunochemical analysis. TEM observation were performed on L4 vertebral bodies of randomly selected rats: one in the control group and two in the OVX group at each time point. Differences in bone marrow perfusion and bone marrow microenvironment may exist at different points along the lumbar spine with varying BMD values [21, 22], The mismatch between imaging and TEM examination may affect the results. Third, only one semi-quantitative DCE-MRI parameter was selected to compare with the quantitative DCE-MRI parameters. However, E_max_ is one of the most common semi-quantitative indexes in the measurement of perfusion because of its relative stability and repeatability.

## Conclusion

In comparison with semi-quantitative DCE-MRI, the quantitative DCE-MRI parameter K^trans^ is a sensitive and accurate index for demonstrating early reduced bone marrow perfusion in the OP.

## Abbreviations

BMD: Bone mineral density
DCE-MRI: Dynamic contrast-enhanced magnetic resonance imaging
DXA: Dual-energy X-ray absorptiometry
E_max_: Maximum enhancement
L5: The fifth lumbar vertebra
micro-CT: Micro-computed tomography
MVD: Microvessel density
OP: Osteoporosis
OVX: Ovariectomy
ROI: Region of interest
TEM: Transmission electron microscopy
TIC: Time–signal intensity curve
VEC: "Vascular endothelial cell
